# Supplementary material for: A Critical View of the Application of the APEX Software (Aqueous Photochemistry of Environmentally-Occurring Xenobiotics) to Predict Photoreaction Kinetics in Surface Freshwaters
Source: Molecules. 2019 Dec 18;25(1):9. doi: 10.3390/molecules25010009 (PMC7017383; doi:10.3390/molecules25010009)
Supplement: Supplementary file 1 [file molecules-25-00009-s001.zip › 3.2.4_gcc-4.4.0.zip!/3.2.4_gcc-4.4.0/3.2.4_gcc-4.4.0/doc/octave/html/interpreter/Invoking-Octave-from-the-Command-Line.html]

Invoking Octave from the Command Line - Untitled


Next: Quitting Octave,
Up: Getting Started


---

### 2.1 Invoking Octave from the Command Line

Normally, Octave is used interactively by running the program
octave without any arguments. Once started, Octave reads
commands from the terminal until you tell it to exit.

You can also specify the name of a file on the command line, and Octave
will read and execute the commands from the named file and then exit
when it is finished.

You can further control how Octave starts by using the command-line
options described in the next section, and Octave itself can remind you
of the options available. Type octave --help to display all
available options and briefly describe their use (octave -h is a
shorter equivalent).

- Command Line Options- Startup Files
